# Micronutrient Antioxidants and Nonalcoholic Fatty Liver Disease

**DOI:** 10.3390/ijms17091379

**Published:** 2016-08-23

**Authors:** Guanliang Chen, Yinhua Ni, Naoto Nagata, Liang Xu, Tsuguhito Ota

**Affiliations:** Department of Cell Metabolism and Nutrition, Brain/Liver Interface Medicine Research Center, Kanazawa University, Kanazawa, Ishikawa 920-8640, Japan; guanliangc@gmail.com (G.C.); shali0145@gmail.com (Y.N.); nnagata@staff.kanazawa-u.ac.jp (N.N.); liangxu1023@gmail.com (L.X.)

**Keywords:** nonalcoholic fatty liver disease (NAFLD), nonalcoholic steatohepatitis (NASH), antioxidants, insulin resistance, inflammation, fibrosis, vitamin D, vitamin E, astaxanthin

## Abstract

Nonalcoholic fatty liver disease (NAFLD) is one of the most important chronic liver diseases worldwide and has garnered increasing attention in recent decades. NAFLD is characterized by a wide range of liver changes, from simple steatosis to nonalcoholic steatohepatitis, cirrhosis, and hepatocellular carcinoma. The blurred pathogenesis of NAFLD is very complicated and involves lipid accumulation, insulin resistance, inflammation, and fibrogenesis. NAFLD is closely associated with complications such as obesity, diabetes, steatohepatitis, and liver fibrosis. During the progression of NAFLD, reactive oxygen species (ROS) are activated and induce oxidative stress. Recent attempts at establishing effective NAFLD therapy have identified potential micronutrient antioxidants that may reduce the accumulation of ROS and finally ameliorate the disease. In this review, we present the molecular mechanisms involved in the pathogenesis of NAFLD and introduce some dietary antioxidants that may be used to prevent or cure NAFLD, such as vitamin D, E, and astaxanthin.

## 1. Introduction

Nonalcoholic fatty liver disease (NAFLD) is characterized as the accumulation of triglyceride (TG)-based fat of more than 5% to 10% of the liver weight in the absence of superfluous alcohol consumption. NAFLD may progress to end-stage liver disease and has become a worldwide health concern [1]. By the year 2015, the conservative estimate of the global incidence of NAFLD was 25.24%, suggesting that more than 1 billion people have NAFLD worldwide [2]. Global epidemiologic research has suggested that males are more susceptible to developing NAFLD than are females, while a higher prevalence of NAFLD is often correlated with countries that have a higher economic status [3,4,5]. Current data show that the prevalence of NAFLD in the US is associated with the prevalence of obesity (>30%), which means that one-third of adults in the US may have already developed NAFLD [6]. The information explosion and improved global economics in the last few decades have driven societal modernization, making high-energy Western food popular worldwide; this has become the leading reason for the increasing prevalence of NAFLD in Eastern and developing countries. Studies in Japan, Korea, and China have shown that the risk of an NAFLD diagnosis has increased by three- to four-fold over the past three years, and the rate of NAFLD is similar to that in Western countries, ranging from 15% to 30% [7,8]. These previous studies found that in regions which traditional diets and lifestyles are maintained, such as rural India and sub-Saharan Africa, the incidence of NAFLD is much lower than in any of the above-mentioned regions. This indicates that excess calorie intake and more comfortable lifestyles involving less exercise may be important contributors to the development of fatty liver disease [9,10,11].

NAFLD is characterized by a wide histologic spectrum of liver damage, including, but not limited to, simple steatosis, nonalcoholic steatohepatitis (NASH), hepatic fibrosis, and cirrhosis [12]. NASH is described as steatosis combined with inflammation and ballooning and has become the second leading hepatic disease resulting in liver transplantation in the US [13]. According to published data, approximately one-third of adults in the US who have NAFLD also have NASH, and 30% of these individuals have the potential to progress to advanced cirrhosis, hepatocellular carcinoma (HCC), and liver-related mortality [14,15]. On the other hand, NAFLD is frequently associated with obesity and type-2 diabetes mellitus (T2DM) because they have similar features of metabolic syndrome such as chronic inflammation and insulin resistance [16]. Hyperglycemia, a typical symptom of T2DM, and a high body mass index (BMI), the characteristic of obesity, are the representative risk factors for NAFLD [4,17,18]. Published reports state that 50% to 73% of patients with end-stage liver disease, such as cryptogenic cirrhosis, have an obese-category BMI or diabetes [19,20]. Importantly, the prevalence of obese children affected by NAFLD is 10- to 20-fold higher than that of lean children [21,22].

Attempts to cure NAFLD and its subsequent complications have been ongoing for decades. Researchers worldwide have concentrated on revealing the potential molecular mechanisms of NAFLD formation and its progression to NASH, cirrhosis, and HCC. Integrative approaches help to clarify the pathogenesis of NAFLD and include an understanding of the apparent hepatic accumulation of excess fat and lipids of diverse sources, abstruse crosstalk among daily diet elements, the composition of the gut microbiota, and the role of epigenetics on the background of weight gain and obesity [23]. However, the numerous laboratory and clinical studies have made NAFLD a complicated issue. This condition is not simply a consequence of metabolic syndrome and insulin resistance; it also involves many complications such as inflammation, chronic kidney disease, and cardiovascular disease. Recent efforts have focused on identifying novel potential targets that can serve as indirect therapies for NAFLD during its progression. In this review, we discuss various factors related to the pathogenesis of NAFLD and introduce some micronutrient antioxidants that may be used in NAFLD prevention and therapy.

## 2. Pathogenesis of Nonalcoholic Fatty Liver Disease (NAFLD)

**Figure 1:**
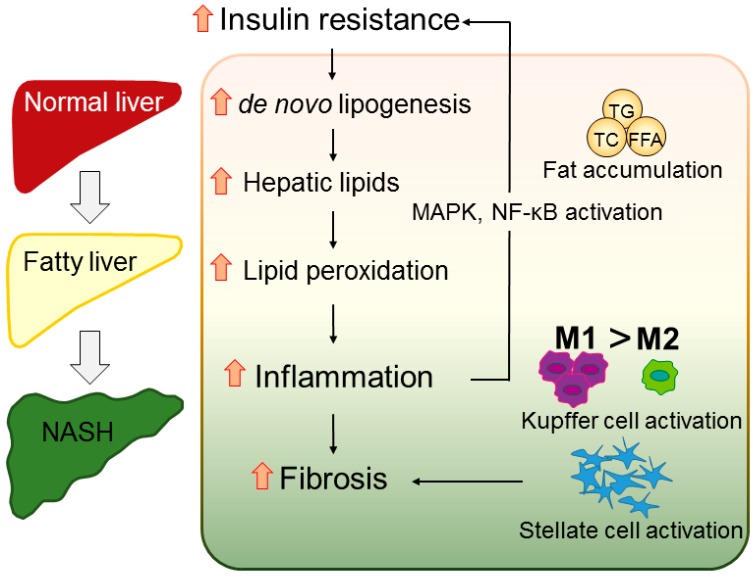
Hypothetic mechanism of nonalcoholic fatty liver disease/nonalcoholic steatohepatitis (NAFLD/NASH) progression. Excessive intake of excess calories and fat results in accumulation of triglycerides, total cholesterol, and free fatty acids, inducing hepatic steatosis. The overload of liver lipids enhances lipid peroxidation, which induces the production of reactive oxygen species and steatohepatitis. Hepatic inflammation activates the mitogen-activated protein kinase pathway and nuclear factor-κB, resulting in insulin resistance. Insulin resistance also promotes de novo lipogenesis, forcing the healthy liver to develop NASH. The inflammation also recruits Kupffer cells and polarizes M1 macrophages, activating hepatic stellate cells and finally leading to liver fibrosis. TG, triglycerides; TC, total cholesterol; FFA, free fatty acids; MAPK, mitogen-activated protein kinase; NF-κB, nuclear factor-κB.

The mainstream concept of NAFLD is the “multiple parallel hits” hypothesis, which developed from the two-hit theory proposed by Day et al. [24] in 1998. The two-hit theory states that a high-fat diet or diabetes-induced steatosis (the first hit) will make the liver more sensitive to other risk factors related to oxidative stress and induce severe lipid peroxidation (the second hit). The multiple parallel hits theory states that NAFLD is a more comprehensive effect of diverse factors, such as endoplasmic reticulum stress, chemokines and cytokines, and innate immunity, than a simple effect of one or two factors, which may explain why NAFLD is also observed in lean people [25] (Figure 1).

### 2.1. Obesity

Many studies have proposed that obese patients have a greater risk of developing NAFLD (75%–100%) than the general population because their higher serum alanine aminotransferase (ALT) and aspartate aminotransferase (AST) concentrations reflect liver injury caused by hepatic steatosis [26]. The leading cause of hepatic fat accumulation in the pathogenesis of NAFLD is overactive fatty acid circulation with transcription factor disorders induced by lipogenesis and fatty acid synthesis, as well as fatty acid oxidation [27]. Adipocytes, as important mediators of systemic lipid storage and adipokine release, gather the excessive fatty acids as TGs in tissues, which then influence processes including lipid metabolism, glucose regulation, and inflammation [28]. The free fatty acids (FFAs) obtained from TG lipolysis are the central source of fat in patients with NAFLD. Along with several other abnormalities, such as hyperglycemia, a low high-density lipoprotein cholesterol level, and hypertension, these FFAs contribute to the development of insulin resistance as a common complication of NAFLD [29]. The risk of lipolysis in visceral adipose tissue is higher than that in subcutaneous adipose tissue, which causes patients with visceral fat accumulation-induced central obesity to be universally insulin resistant and much more likely to develop NAFLD secondary to their increasing FFA content [19].

Adiponectin is an adipose-specific secretory adipokine that can induce FFA oxidation and lipid transfer to inhibit FFA accumulation with its corresponding receptor in the liver [30]. Adiponectin is a link between adipose tissue and whole-body glucose metabolism, which can affect hepatic insulin sensitivity [31]. Recent studies have found that the serum adiponectin level is much lower in patients with, than without, NAFLD [32]. In addition to the presence of insulin resistance syndrome, metabolic disturbances appear to be present, as evidenced by excess ectopic fat and dysfunctional adipose tissue [33]. Hypoadiponectinemia, a typical trait of NAFLD, suggests that adiponectin, as an antagonist of tumor necrosis factor α (TNF-α), has anti-lipogenic and anti-inflammatory effects that can protect the liver from damage by maintaining the balance between pro-inflammatory and anti-inflammatory cytokines in hepatocytes [34]. Furthermore, the serum adiponectin concentration coupled with the waist-to-hip ratio and AST/ALT ratio could serve as a novel tool with which to diagnose advanced fibrosis of NAFLD, suggesting that increasing adiponectin levels may be a new therapeutic method for inflammation and fibrosis in patients with NAFLD [35]. Interestingly, because adiponectin is secreted mostly by subcutaneous fat rather than visceral fat, hypoadiponectinemia may also help to explain why patients with central obesity more commonly develop insulin resistance among patients with NAFLD [30] (Table 1).

### 2.2. Diabetes

#### 2.2.1. Diabetes to NAFLD

As a consequence of obesity and low adiponectin production induced by long-term oversupply of calories, hyperlipidemia and insulin resistance are frequently found in patients with NAFLD, considerably strengthening the association between this metabolic syndrome and diabetes. A few studies have found a strong link between insulin-dependent diabetes mellitus (also known as type-1 diabetes mellitus) and NASH in adolescents [57,58]. Many other studies have focused on the relationship between T2DM and NAFLD, which is complex and bidirectional [59]. A clinical study of general health examinations in Japan found that about 29% of middle-aged Japanese adults have NAFLD and that a substantial proportion of them also had insulin resistance syndrome [60]. With their impaired glucose metabolism and abnormally elevated TG concentration, patients with concurrent T2DM and NAFLD have a greater risk of progression to NASH [61,62].

#### 2.2.2. NAFLD to Diabetes

Likewise, another recent study found that NAFLD also increases the risk of developing T2DM. Using liver ultrasound technology and hepatic biopsy, a study in the US indicated that the incidence of diabetes is three-fold higher in patients with NAFLD than in the general population [63]. Another salient characteristic of NAFLD is hepatic steatosis, which causes redundant nonesterified fatty acid as an intrinsic defect and induces peripheral insulin resistance and endocrine over-reaction, the typical features of T2DM [64]. In patients with NAFLD with aberrant glucose metabolism, the insulin sensitivity is impaired in adipose tissue, liver, and muscle, but only adipose tissue glucose intolerance will exacerbate T2DM [65]. Due to the precise relationship between NAFLD and diabetes, the most effective therapy for NAFLD appears to be the indirect method of improving abnormal hepatic lipid metabolism by ameliorating glucose dysregulation and enhancing insulin sensitivity [59].

The prevalence of some other fatal diseases is also heightened in populations with these two complications. In patients with diabetes, for instance, the highest standardized mortality ratio is associated with liver cirrhosis; hepatic cirrhosis also elevates the risk of death from cardiovascular disease in patients with diabetes [66]. A former study demonstrated that more than 34% of patients with diabetes have NAFLD and that the combination of these two diseases enhanced the risk of death from malignancy [67]. Frequently, NAFLD also increases the risk of developing microvascular diseases such as chronic kidney disease in patients with T2DM. The increased γ-glutamyltransferase concentrations caused by NAFLD may be associated with some severe subclinical renal disease and the risk of T2DM [68]. A recent study in Italy involving a large number of participants estimated that the prevalence of chronic kidney disease in diabetic patients with NAFLD is 60% higher than that in their counterparts without NAFLD [69].

### 2.3. Inflammation

The morphological mitochondrial alterations in patients with NAFLD, including cholesterol and FFA accumulation, induce oxidative stress and the formation of reactive oxygen species (ROS) [70]. These ROS, in turn, instigates lipid peroxidation, which leads to the generation of aldehyde byproducts such as malondialdehyde, triggering TNF-α-regulated liver damage [71]. Thus, the TNF-α-induced increase in inflammatory activity against a background of abnormal lipid metabolism and resultant lipotoxicity is considered to lead to the whole spectrum of NAFLD pathologies [25,72]. The stress-related protein kinase cascade Jun N-terminal kinase (JNK) is induced by TNF-α and phosphorylates the proto-oncogene c-Jun, stimulating epidermal growth factor to accelerate proliferation. Oxidant-sensitive transcription factors such as nuclear factor-κB (NF-κB) are then also invigorated by ROS, up-regulating the expression of cytokines including interleukin-6 (IL-6) and IL-1β [73]. Growing evidence suggests that activation of JNK, NF-κB, and proinflammatory cytokines is central to interposing insulin resistance through inhibition of insulin receptor signaling and suppression of organ insulin sensitivity [74,75]. Therefore, as the antagonist of adiponectin, the TNF-α-induced activation of proinflammatory cytokines and hypoadiponectinemia with glucose intolerance can assist in distinguishing patients with NASH from those with simple steatosis [76].

The immune system is typically involved in the progression of NAFLD. The activation of cytochrome P450 isoenzymes in association with TNF-α mitochondrial effects promotes whole-organism fatty acid oxidation. This causes oxidative stress secondary to increased ROS, which thereafter induces immune responses in patients with NAFLD. These reactions are associated with the formation of advanced disease [77]. Immune cells, including T lymphocytes (T cells), natural killer T cells (NKT cells), and macrophages, impact on NAFLD progression to NASH. Some former reports have reported that CD8^+^ T-cell infiltration is present in the epididymal adipose tissue of obese mice fed a high-fat diet, which induces adipose tissue inflammation and systemic insulin resistance. In contrast, collaboration of CD4^+^ T cells with CD3^+^ T cells can potently reduce obesity and insulin resistance syndromes that cause atherosclerotic plaques, which may be therapeutically beneficial in patients with diabetes [78,79]. In addition, as the components of adaptive immunity, NKT cell numbers are low in mice with obesity-induced liver injury, suggesting that NKT cells may play pathophysiologic roles in steatosis and fat-induced inflammation [80,81].

Resident macrophages and infiltrating monocytes are essential ingredients of innate immunity. These cells are associated with inflammation and subsequent tissue renovation through clearing necrotic/apoptotic cells, recruiting and activating myofibroblasts, and regulating the secretion of anti-inflammatory cytokines and growth factors such as IL-10 and TGF-β [82,83]. In tissues, monocytes and macrophages are distinguished by their substantial diversity and plasticity. Mononuclear phagocytes can respond to environmental irritations by acquiring different functional phenotypes, while macrophages can be divided into classically-activated macrophages (M1 macrophages) and alternatively-activated macrophages (M2 macrophages) with IL-4 stimulation according to the presence of Toll-like receptor ligands such as lipopolysaccharides, which are directly affected by microbial stimuli [84,85]. Previous in vitro and in vivo studies have concluded that the imbalance of polarization between M1/M2 phenotypic macrophages will induce chronic inflammation, various infections, systemic allergies, cancer, obesity, and diabetes, as well as NAFLD [84,86]. A promising therapy for NAFLD was recently identified: specific macrophage-targeted treatment. This therapy can help to restrain the polarization of M1 macrophages/Kupffer cells (KCs) and/or induce the protective phenotype of M2 macrophages/KCs [87,88].

Chemokines are a family of cytokines that activate leukocytes and play important roles during the progression of inflammation [16]. Many published reports have investigated the effects of chemokines in acute inflammation and chronic monocyte- or lymphocyte-predominant inflammation [89]. For instance, the expression of monocyte chemoattractant protein 1 (MCP-1), also known as C-C chemokine ligand 2 (CCL2), is up-regulated in adipose tissue secondary to macrophage infiltration [90]. Combined with its receptor, C-C chemokine receptor type 2 (CCR2), the MCP-1–CCR2 system is closely associated with hepatic steatosis and insulin resistance in obese patients [91,92]. Our former study also revealed that CCR5 inhibition may become a novel therapeutic target for patients with glucose intolerance and T2DM through the regulation of macrophage recruitment and the response of M1/M2 macrophage polarization to inflammation [93].

### 2.4. Fibrosis

As a crucial response to chronic injury and macrophage activation, fibrosis indisputably plays a key role during the progression of NAFLD to NASH [94]. In the course of hepatic fibrosis, the trans-differentiation of hepatic stellate cells (HSCs) into myofibroblasts (known as activated HSCs) produces extracellular matrix components and causes extensive scarring with the formation of abundant dying necrotic cells and debris [95,96]. The KCs and recruited macrophages then guide phagocytosis of the dying necrotic cells and debris; this can induce the formation of TGF-β which also accelerates fibrosis [97,98,99]. Furthermore, monocytes and macrophages expressing chemokine receptors, including CCR2 and CCR5, are thought to be involved in the activation and migration of HSCs through TGF-β to promote liver fibrosis [100,101].

## 3. Micronutrients and NAFLD/Nonalcoholic Steatohepatitis (NASH)

In addition to the established treatment involving sustained weight loss by increased physical training and diet control, there is no consensus on the most effective pharmacological therapies for NAFLD/NASH because the detailed pathophysiology of NAFLD remains incompletely understood [102,103,104]. One popular approach involves the use of components for secondary therapy of complications, such as hepatic fat accumulation, insulin resistance, inflammation, and fibrosis. For example, pioglitazone and metformin, common treatments for glucose intolerance, can enhance insulin sensitivity in patients with NAFLD/NASH; however, other histological features, such as fibrosis, are not significantly influenced [105,106]. Pirfenidone, a therapeutic agent used for fibrosis, can reduce the serum ALT and AST concentrations and has anti-fibrotic and anti-inflammatory properties that help to reverse liver injury [107,108]. Not only do these potential treatments require the development of an additional therapeutic plan but their molecular mechanisms during treatment also remain unclear. Thus, most recommendations encourage the consumption of micronutrients, which have anti-oxidative and anti-inflammatory effects, to prevent and treat NAFLD [109,110].

### 3.1. Vitamin D

Previous studies reported that the serum vitamin D levels of the patients with NAFLD/NASH were lower than those without diseases, suggesting that NAFLD might cause vitamin D deficiency in these patients [111,112]. Rhee et al. [113] observed that subjects with higher plasma vitamin D level showed a remarkably decreased risk of NAFLD to low level groups. However, recent research found the connection between NAFLD and vitamin D in both adult and children populations, that vitamin D concertation is inversely associated with NAFLD/NASH and fibrosis, independent of metabolic syndrome, insulin resistance, liver fat accumulation or severity of NASH [114,115,116]. As a secosteroid associated with calcium homeostasis, the functions of vitamin D on immune modulation, cell differentiation and proliferation, and the inflammatory response have already be confirmed [117]. For instance, vitamin D deficiency would activate Toll-like receptors, resulting in severe liver inflammation and induction of oxidative stress. Vitamin D supplements could reverse the inflammation caused by NAFLD-related hepatic injury by inhibiting monocyte activation and TNF-α and IL-1 expression. [118,119]. Further studies are still needed to identify whether vitamin D has benefits on NAFLD/NASH therapy.

### 3.2. Vitamin E

As a common antioxidant, vitamin E has been used as a therapeutic component for NAFLD by inhibiting ROS production during the development of steatohepatitis. One study showed that, compared with the control group, 43% of patients with NASH showed clinical improvement with significant reductions in their ALT and AST levels and lobular inflammation after treatment with vitamin E [105]. Similar effects were reported in a clinical study in which vitamin E ameliorated NASH by decreasing the ALT concentration and histological activity, and promoted weight control [120]. More generally, vitamin E is often used with other therapeutic methods, such as comprehensive weight reduction programs, leading to weight loss and normalized serum enzyme concentrations in obese children with NASH [121]. A prospective, double-blind, randomized, placebo-controlled trial observed that six months of combination treatment with vitamins E and C alleviated fibrosis in patients with NASH without improvement in the necroinflammatory activity or ALT concentration [122]. Nevertheless, some studies have shown that vitamin E is not superior to placebo in ameliorating NAFLD or, even worse, that daily supplementation of vitamin E may increase the risk of prostate cancer [106,123].

### 3.3. Astaxanthin

Astaxanthin is a xanthophyll carotenoid that is abundant in many microorganisms and marine animals including yeast, salmon, shrimp, and crayfish, as well as a special green microalga, *Haematococcus pluvialis* [124]. As a novel natural antioxidant, astaxanthin is cultivated at an industrial scale for its health effects, including anticancer activity, immune modulation, and cardiovascular disease prevention. It is also much more effective than vitamin E at protecting mitochondria from lipid peroxidation in liver cells [124,125,126,127]. In addition to its anti-diabetic and anti-inflammatory effects, astaxanthin can prevent the up-regulation of glutamate transaminase activity and thiobarbituric acid reactive substances from carbon tetrachloride-induced lipid peroxidation as well as increase the levels of glutathione and superoxide dismutase in rat liver [128]. Moreover, astaxanthin can protect against diet-induced obesity by blocking increases in body weight and adipose tissue, reducing lipid accumulation, and ameliorating oxidative stress-induced insulin resistance through enhancement of insulin signals and inhibition of extracellular signal-regulated kinase (ERK) and JNK phosphorylation [129,130]. Moreover, astaxanthin can reduce the cellular accumulation of ROS and block TGF-β signaling, suppressing activation of the Smad3 pathway in HSCs, consequently preventing the development of liver fibrosis [131,132].

**Figure 2:**
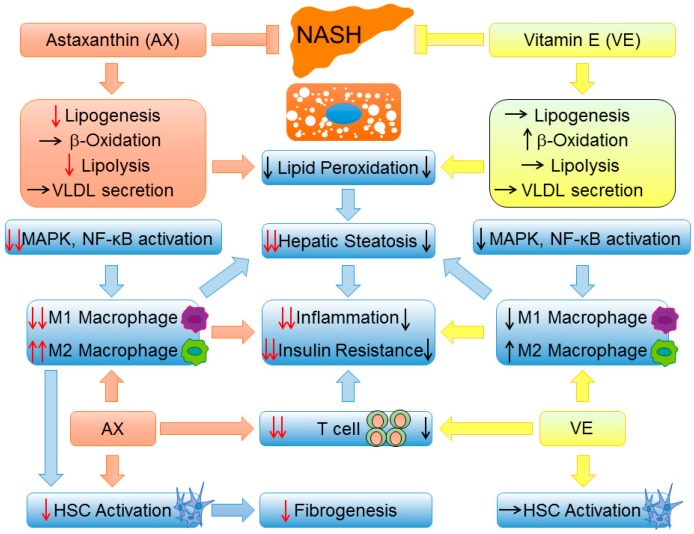
A brief comparison of NAFLD/NASH prevention and therapy between astaxanthin and vitamin E. First, astaxanthin is more effective than vitamin E in improving steatosis by suppressing lipid accumulation. Second, astaxanthin is superior to vitamin E with respect to suppressing the MAPK pathway and NF-κB activation and induces a strong shift of M2 macrophage polarization, which ultimately reverses hepatic steatosis, inflammation, and insulin resistance. Most importantly, as a result of M1/M2 transformation, astaxanthin can reduce hepatic stellate cell (HSC) activation and ameliorate hepatic fibrosis. Black arrow: ↑: induction, ↓: inhibition, →: no change; Red arrow: ↓: inhibition compared with vitamin E, ↑↑/↓↓: more significant induction/inhibition effect compared with vitamin E.

In our previous study, we examined the preventative and therapeutic effects of astaxanthin, both in vivo and in vitro [133]. Astaxanthin was more effective than vitamin E in reducing liver lipid accumulation, ameliorating insulin resistance, and protecting against inflammation and fibrosis in mice with lipotoxicity-induced NASH. For instance, astaxanthin decreased the concentrations of TG, total cholesterol, nonesterified fatty acids, ALT, and AST, preventing the transformation of simple steatosis to NASH in obese mice. Additionally, astaxanthin inhibited activation of the JNK/p38 mitogen-activated protein kinases (MAPK) pathway and NF-κB, reduced the production of T cells and macrophages, and induced an M2-dominant shift in macrophages/KCs to reverse inflammation and glucose intolerance. Moreover, astaxanthin significantly attenuated hepatic fibrosis by down-regulating the expression of fibrogenic genes and decreasing the hydroxyproline content. On the other hand, compared with placebo, astaxanthin treatment reduced the severity of steatosis and tended to alleviate lobular inflammation, resulting in marked improvement of hepatic steatosis. Overall, considering the above-described benefits, astaxanthin may become a promising agent in the prevention or treatment of NAFLD/NASH (Figure 2).

### 3.4. Other Micronutrients

Apart from vitamin D, E, and astaxanthin, some other vitamins and carotenoids are also taken into account in the treatment of NAFLD. For instance, the liver is a crucial storage reservoir of vitamin B_12_, and hepatic overexpression of ROS is associated with acute hepatitis, cirrhosis, and HCC. Maternal vitamin B_12_ deficiency will increase the chance that offspring develop adiposity and T2DM, which can be normalized after vitamin B_12_ supplementation [134,135]. β-Cryptoxanthin is a marker of the antioxidant milieu provided by the satsuma mandarin (*Citrus unshiu* Marc.), and its shortage in blood may induce lipid peroxidation and oxidative DNA damage [136]. Our previous studies also revealed that β-cryptoxanthin prevents progression of NAFLD by reducing fat accumulation, reversing insulin resistance, activating M2-dominant polarization in macrophages/KCs, and suppressing oxidative stress and fibrosis in mouse models of lipotoxicity-induced NASH [137,138].

Other micronutrients such as obeticholic acid and silymarin, which are derived from natural substances, also have therapeutic potential according to some reports. Obeticholic acid can ameliorate NAFLD/NASH by increasing insulin sensitivity and reducing liver enzyme levels and fibrosis [139]. Silymarin, which is an extract of the milk thistle plant (*Silybum marianum*), can attenuate hepatic lipid metabolism and oxidative stress in mice with NAFLD [140].

## 4. Conclusions

Increasing attention has been focused on NAFLD, which may become the most universal and severe chronic liver disease worldwide in the next few decades. The pathogenetic mechanisms of NAFLD are complex and associated with various complications, such as obesity, T2DM, hepatitis, and fibrosis. Current research has shown that overproduction of ROS and changes in the contents of some central factors including adiponectin, chemokines, TNF-α and TGF-β may be the main promoters of NAFLD development. Novel preventative and therapeutic strategies include the development of micronutrient antioxidants that resist oxidative stress and normalize various factors. Maintaining physical exercise habits with healthy dietary supplements, including these micronutrients, for example, the Mediterranean diet, which contains silymarin phytosome complex and vitamin E, can be a promising method for the management of NAFLD [141,142]. Further studies are necessary to obtain better knowledge of the pathophysiology of NAFLD and, therefore, the potential role of micronutrients in the prevention and treatment of NAFLD.

## Figures and Tables

**Table 1:** Major adipokines involved in nonalcoholic fatty liver disease (NAFLD) pathogenesis.

Adipokines	Function
Adiponectin	Anti-inflammatory, improve insulin sensitivity, prevent lipid accumulation, attenuate fibrosis, inhibit tumor necrosis factor (TNF-α) synthesis and/or release [36,37,38,39,40]
Leptin	Prevent lipid accumulation, amplify inflammation, induce fibrosis, increase TNF-α concentration [41,42,43,44]
TNF-α	Promote inflammation, induce lipid accumulation and insulin resistance, pro-fibrotic effect [45,46,47,48]
Resistin	Cause insulin resistance, reduce interleukin 6 (IL-6) secretion, participate in liver fibrogenesis [49,50,51,52,53]
IL-6	Suppress oxidative stress and prevent mitochondrial dysfunction [54,55,56]

## References

[1] Angulo P. (2002). Nonalcoholic fatty liver disease. N. Engl. J. Med..

[2] Younossi Z.M., Koenig A.B., Abdelatif D., Fazel Y., Henry L., Wymer M. (2015). Global epidemiology of non-alcoholic fatty liver disease–meta-analytic assessment of prevalence, incidence and outcomes. Hepatology.

[3] Zhu J.Z., Dai Y.N., Wang Y.M., Zhou Q.Y., Yu C.H., Li Y.M. (2015). Prevalence of nonalcoholic fatty liver disease and economy. Dig. Dis. Sci..

[4] Liu C.J. (2012). Prevalence and risk factors for non-alcoholic fatty liver disease in Asian people who are not obese. J. Gastroenterol. Hepatol..

[5] Sanyal A.J. (2002). Aga technical review on nonalcoholic fatty liver disease. Gastroenterology.

[6] Loomba R., Sanyal A.J. (2013). The global NAFLD epidemic. Nat. Rev. Gastroenterol. Hepatol..

[7] Farrell G.C., Wong V.W., Chitturi S. (2013). Nafld in Asia—As common and important as in the west. Nat. Rev. Gastroenterol. Hepatol..

[8] Fan J.G., Jia J.D., Li Y.M., Wang B.Y., Lu L.G., Shi J.P., Chan L.Y. (2011). Guidelines for the diagnosis and management of nonalcoholic fatty liver disease. J. Dig. Dis..

[9] Singh S., Nayak S., Swain M., Rout N., Mallik R., Agrawal O., Meher C., Rao M. (2003). Prevalence of nonalcoholic fatty liver disease in coastal eastern india: A preliminary ultrasonographic survey. Trop. Gastroenterol..

[10] Amarapurkar D.N., Hashimoto E., Lesmana L.A., Sollano J.D., Chen P.J., Goh K.L. (2007). How common is non-alcoholic fatty liver disease in the Asia-Pacific region and are there local differences?. J. Gastroenterol. Hepatol..

[11] Onyekwere C.A., Ogbera A.O., Balogun B.O. (2011). Non-alcoholic fatty liver disease and the metabolic syndrome in an urban hospital serving an African community. Ann. Hepatol..

[12] Marcuccilli M., Chonchol M. (2016). Nafld and chronic kidney disease. Int. J. Mol. Sci..

[13] Wong R.J., Aguilar M., Cheung R., Perumpail R.B., Harrison S.A., Younossi Z.M., Ahmed A. (2015). Nonalcoholic steatohepatitis is the second leading etiology of liver disease among adults awaiting liver transplantation in the united states. Gastroenterology.

[14] Marchesini G., Bugianesi E., Forlani G., Cerrelli F., Lenzi M., Manini R., Natale S., Vanni E., Villanova N., Melchionda N. (2003). Nonalcoholic fatty liver, steatohepatitis, and the metabolic syndrome. Hepatology.

[15] Caldwell S., Argo C. (2010). The natural history of non-alcoholic fatty liver disease. Dig. Dis..

[16] Xu L., Kitade H., Ni Y., Ota T. (2015). Roles of chemokines and chemokine receptors in obesity-associated insulin resistance and nonalcoholic fatty liver disease. Biomolecules.

[17] Fan J.G., Saibara T., Chitturi S., Kim B.I., Sung J.J., Chutaputti A. (2007). What are the risk factors and settings for non-alcoholic fatty liver disease in Asia-Pacific?. J. Gastroenterol. Hepatol..

[18] Aykut U.E., Akyuz U., Yesil A., Eren F., Gerin F., Ergelen R., Celikel C.A., Yilmaz Y. (2014). A comparison of fibrometer nafld score, nafld fibrosis score, and transient elastography as noninvasive diagnostic tools for hepatic fibrosis in patients with biopsy-proven non-alcoholic fatty liver disease. Scand. J. Gastroenterol..

[19] Angulo P. (2007). GI epidemiology: Nonalcoholic fatty liver disease. Aliment. Pharmacol. Ther..

[20] Clark J.M., Diehl A.M. (2003). Nonalcoholic fatty liver disease: An underrecognized cause of cryptogenic cirrhosis. JAMA.

[21] Saviano M.C., Brunetti F., Rubino A., Franzese A., Vajro P., Argenziano A., Puzziello A., Iannucci M.P. (1997). Liver involvement in obese children (ultrasonography and liver enzyme levels at diagnosis and during follow-up in an italian population). Dig. Dis. Sci..

[22] Tominaga K., Kurata J.H., Chen Y.K., Fujimoto E., Miyagawa S., Abe I., Kusano Y. (1995). Prevalence of fatty liver in Japanese children and relationship to obesity. Dig. Dis. Sci..

[23] Wree A., Broderick L., Canbay A., Hoffman H.M., Feldstein A.E. (2013). From NAFLD to NASH to cirrhosis—New insights into disease mechanisms. Nat. Rev. Gastroenterol. Hepatol..

[24] Day C.P., James O.F. (1998). Steatohepatitis: A tale of two “hits”?. Gastroenterology.

[25] Tilg H., Moschen A.R. (2010). Evolution of inflammation in nonalcoholic fatty liver disease: The multiple parallel hits hypothesis. Hepatology.

[26] Henao-Mejia J., Elinav E., Jin C., Hao L., Mehal W.Z., Strowig T., Thaiss C.A., Kau A.L., Eisenbarth S.C., Jurczak M.J. (2012). Inflammasome-mediated dysbiosis regulates progression of NAFLD and obesity. Nature.

[27] Mendez-Sanchez N., Arrese M., Zamora-Valdes D., Uribe M. (2007). Current concepts in the pathogenesis of nonalcoholic fatty liver disease. Liver Int..

[28] Scherer P.E. (2006). Adipose tissue: From lipid storage compartment to endocrine organ. Diabetes.

[29] Marchesini G., Brizi M., Bianchi G., Tomassetti S., Bugianesi E., Lenzi M., McCullough A.J., Natale S., Forlani G., Melchionda N. (2001). Nonalcoholic fatty liver disease a feature of the metabolic syndrome. Diabetes.

[30] Angulo P. (2006). NAFLD, obesity, and bariatric surgery. Gastroenterology.

[31] Berg A.H., Combs T.P., Du X., Brownlee M., Scherer P.E. (2001). The adipocyte-secreted protein ACRP30 enhances hepatic insulin action. Nat. Med..

[32] Masarone M., Federico A., Abenavoli L., Loguercio C., Persico M. (2014). Non alcoholic fatty liver: Epidemiology and natural history. Rev. Recent Clin. Trials.

[33] Bugianesi E., Pagotto U., Manini R., Vanni E., Gastaldelli A., de Iasio R., Gentilcore E., Natale S., Cassader M., Rizzetto M. (2005). Plasma adiponectin in nonalcoholic fatty liver is related to hepatic insulin resistance and hepatic fat content, not to liver disease severity. J. Clin. Endocrinol. Metab..

[34] Pagano C., Soardo G., Esposito W., Fallo F., Basan L., Donnini D., Federspil G., Sechi L.A., Vettor R. (2005). Plasma adiponectin is decreased in nonalcoholic fatty liver disease. Eur. J. Endocrinol..

[35] Savvidou S., Hytiroglou P., Orfanou-Koumerkeridou H., Panderis A., Frantzoulis P., Goulis J. (2009). Low serum adiponectin levels are predictive of advanced hepatic fibrosis in patients with nafld. J. Clin. Gastroenterol..

[36] Mirza M.S. (2011). Obesity, visceral fat, and nafld: Querying the role of adipokines in the progression of nonalcoholic fatty liver disease. ISRN Gastroenterol..

[37] Anania F.A. (2005). Adiponectin and alcoholic fatty liver: Is it, after all, about what you eat?. Hepatology.

[38] Masaki T., Chiba S., Tatsukawa H., Yasuda T., Noguchi H., Seike M., Yoshimatsu H. (2004). Adiponectin protects LPS-induced liver injury through modulation of TNF-α in KK-AY obese mice. Hepatology.

[39] Kamada Y., Matsumoto H., Tamura S., Fukushima J., Kiso S., Fukui K., Igura T., Maeda N., Kihara S., Funahashi T. (2007). Hypoadiponectinemia accelerates hepatic tumor formation in a nonalcoholic steatohepatitis mouse model. J. Hepatol..

[40] You M., Considine R.V., Leone T.C., Kelly D.P., Crabb D.W. (2005). Role of adiponectin in the protective action of dietary saturated fat against alcoholic fatty liver in mice. Hepatology.

[41] Marra F. (2002). Leptin and liver fibrosis: A matter of fat. Gastroenterology.

[42] Ikejima K., Honda H., Yoshikawa M., Hirose M., Kitamura T., Takei Y., Sato N. (2001). Leptin augments inflammatory and profibrogenic responses in the murine liver induced by hepatotoxic chemicals. Hepatology.

[43] Kakuma T., Lee Y., Higa M., Wang Z.-W., Pan W., Shimomura I., Unger R.H. (2000). Leptin, troglitazone, and the expression of sterol regulatory element binding proteins in liver and pancreatic islets. Proc. Natl. Acad. Sci. USA.

[44] Unger R.H. (2002). Lipotoxic diseases. Annu. Rev. Med..

[45] Hotamisligil G.S., Shargill N.S., Spiegelman B.M. (1993). Adipose expression of tumor necrosis factor-α: Direct role in obesity-linked insulin resistance. Science.

[46] Czaja M.J. (2004). Liver injury in the setting of steatosis: Crosstalk between adipokine and cytokine. Hepatology.

[47] Wellen K.E., Hotamisligil G.S. (2003). Obesity-induced inflammatory changes in adipose tissue. J. Clin. Investig..

[48] Tomita K., Tamiya G., Ando S., Ohsumi K., Chiyo T., Mizutani A., Kitamura N., Toda K., Kaneko T., Horie Y. (2006). Tumour necrosis factor α signalling through activation of kupffer cells plays an essential role in liver fibrosis of non-alcoholic steatohepatitis in mice. Gut.

[49] Jarrar M.H., Baranova A., Collantes R., Ranard B., Stepanova M., Bennett C., Fang Y., Elariny H., Goodman Z., Chandhoke V. (2008). Adipokines and cytokines in non-alcoholic fatty liver disease. Aliment. Pharmacol. Ther..

[50] Rangwala S.M., Rich A.S., Rhoades B., Shapiro J.S., Obici S., Rossetti L., Lazar M.A. (2004). Abnormal glucose homeostasis due to chronic hyperresistinemia. Diabetes.

[51] Satoh H., Nguyen M.A., Miles P.D., Imamura T., Usui I., Olefsky J.M. (2004). Adenovirus-mediated chronic “hyper-resistinemia” leads to in vivo insulin resistance in normal rats. J. Clin. Investig..

[52] Rajala M.W., Obici S., Scherer P.E., Rossetti L. (2003). Adipose-derived resistin and gut-derived resistin-like molecule-β selectively impair insulin action on glucose production. J. Clin. Investig..

[53] Bokarewa M., Nagaev I., Dahlberg L., Smith U., Tarkowski A. (2005). Resistin, an adipokine with potent proinflammatory properties. J. Immunol..

[54] Tsochatzis E.A., Papatheodoridis G.V., Archimandritis A.J. (2009). Adipokines in nonalcoholic steatohepatitis: From pathogenesis to implications in diagnosis and therapy. Mediators Inflamm..

[55] El-Assal O., Hong F., Kim W.-H., Radaeva S., Gao B. (2004). IL-6-deficient mice are susceptible to ethanol-induced hepatic steatosis: IL-6 protects against ethanol-induced oxidative stress and mitochondrial permeability transition in the liver. Cell. Mol. Immunol..

[56] Cressman D.E., Greenbaum L.E., DeAngelis R.A., Ciliberto G. (1996). Liver failure and defective hepatocyte regeneration in interleukin-6-deficient mice. Science.

[57] Rashid M., Roberts E.A. (2000). Nonalcoholic steatohepatitis in children. J. Pediatr. Gastroenterol. Nutr..

[58] Manton N.D., Lipsett J., Moore D.J., Davidson G.P., Bourne A.J., Couper R.T. (2000). Non-alcoholic steatohepatitis in children and adolescents. Med. J. Aust..

[59] Anstee Q.M., Targher G., Day C.P. (2013). Progression of nafld to diabetes mellitus, cardiovascular disease or cirrhosis. Nat. Rev. Gastroenterol. Hepatol..

[60] Jimba S., Nakagami T., Takahashi M., Wakamatsu T., Hirota Y., Iwamoto Y., Wasada T. (2005). Prevalence of non-alcoholic fatty liver disease and its association with impaired glucose metabolism in Japanese adults. Diabet. Med..

[61] Ratziu V., Bellentani S., Cortez-Pinto H., Day C., Marchesini G. (2010). A position statement on NAFLD/NASH based on the EASL 2009 special conference. J. Hepatol..

[62] Ryysy L., Häkkinen A.-M., Goto T., Vehkavaara S., Westerbacka J., Halavaara J., Yki-Järvinen H. (2000). Hepatic fat content and insulin action on free fatty acids and glucose metabolism rather than insulin absorption are associated with insulin requirements during insulin therapy in type 2 diabetic patients. Diabetes.

[63] Williams C.D., Stengel J., Asike M.I., Torres D.M., Shaw J., Contreras M., Landt C.L., Harrison S.A. (2011). Prevalence of nonalcoholic fatty liver disease and nonalcoholic steatohepatitis among a largely middle-aged population utilizing ultrasound and liver biopsy: A prospective study. Gastroenterology.

[64] Bugianesi E., Gastaldelli A., Vanni E., Gambino R., Cassader M., Baldi S., Ponti V., Pagano G., Ferrannini E., Rizzetto M. (2005). Insulin resistance in non-diabetic patients with non-alcoholic fatty liver disease: Sites and mechanisms. Diabetologia.

[65] Ortiz-Lopez C., Lomonaco R., Orsak B., Finch J., Chang Z., Kochunov V.G., Hardies J., Cusi K. (2012). Prevalence of prediabetes and diabetes and metabolic profile of patients with nonalcoholic fatty liver disease (NAFLD). Diabetes Care.

[66] de Marco R., Locatelli F., Zoppini G., Verlato G., Bonora E., Muggeo M. (1999). Cause-specific mortality in type 2 diabetes. The verona diabetes study. Diabetes Care.

[67] Adams L.A., Harmsen S., St Sauver J.L., Charatcharoenwitthaya P., Enders F.B., Therneau T., Angulo P. (2010). Nonalcoholic fatty liver disease increases risk of death among patients with diabetes: A community-based cohort study. Am. J. Gastroenterol..

[68] Chang Y., Ryu S., Sung E., Woo H.Y., Oh E., Cha K., Jung E., Kim W.S. (2008). Nonalcoholic fatty liver disease predicts chronic kidney disease in nonhypertensive and nondiabetic Korean men. Metabolism.

[69] Targher G., Bertolini L., Rodella S., Zoppini G., Lippi G., Day C., Muggeo M. (2008). Non-alcoholic fatty liver disease is independently associated with an increased prevalence of chronic kidney disease and proliferative/laser-treated retinopathy in type 2 diabetic patients. Diabetologia.

[70] Pessayre D., Mansouri A., Fromenty B.V. (2002). Mitochondrial dysfunction in steatohepatitis. Am. J. Physiol. Gastrointest. Liver Physiol..

[71] Mari M., Caballero F., Colell A., Morales A., Caballeria J., Fernandez A., Enrich C., Fernandez-Checa J.C., Garcia-Ruiz C. (2006). Mitochondrial free cholesterol loading sensitizes to TNF- and FAS-mediated steatohepatitis. Cell Metab..

[72] Feldstein A.E., Werneburg N.W., Canbay A., Guicciardi M.E., Bronk S.F., Rydzewski R., Burgart L.J., Gores G.J. (2004). Free fatty acids promote hepatic lipotoxicity by stimulating TNF-α expression via a lysosomal pathway. Hepatology.

[73] Tilg H., Diehl A.M. (2000). Cytokines in alcoholic and nonalcoholic steatohepatitis. N. Engl. J. Med..

[74] Hotamisligil G.S. (2006). Inflammation and metabolic disorders. Nature.

[75] Liu Q., Bengmark S., Qu S. (2010). The role of hepatic fat accumulation in pathogenesis of non-alcoholic fatty liver disease (NAFLD). Lipids Health Dis..

[76] Hui J.M., Hodge A., Farrell G.C., Kench J.G., Kriketos A., George J. (2004). Beyond insulin resistance in NASH: TNF-α or adiponectin?. Hepatology.

[77] Albano E., Mottaran E., Vidali M., Reale E., Saksena S., Occhino G., Burt A.D., Day C.P. (2005). Immune response towards lipid peroxidation products as a predictor of progression of non-alcoholic fatty liver disease to advanced fibrosis. Gut.

[78] Nishimura S., Manabe I., Nagasaki M., Eto K., Yamashita H., Ohsugi M., Otsu M., Hara K., Ueki K., Sugiura S. (2009). Cd8^+^ effector T cells contribute to macrophage recruitment and adipose tissue inflammation in obesity. Nat. Med..

[79] Winer S., Chan Y., Paltser G., Truong D., Tsui H., Bahrami J., Dorfman R., Wang Y., Zielenski J., Mastronardi F. (2009). Normalization of obesity-associated insulin resistance through immunotherapy. Nat. Med..

[80] Li Z., Soloski M.J., Diehl A.M. (2005). Dietary factors alter hepatic innate immune system in mice with nonalcoholic fatty liver disease. Hepatology.

[81] Maher J.J., Leon P., Ryan J.C. (2008). Beyond insulin resistance: Innate immunity in nonalcoholic steatohepatitis. Hepatology.

[82] Duffield J.S., Forbes S.J., Constandinou C.M., Clay S., Partolina M., Vuthoori S., Wu S., Lang R., Iredale J.P. (2005). Selective depletion of macrophages reveals distinct, opposing roles during liver injury and repair. J. Clin. Investig..

[83] Liaskou E., Zimmermann H.W., Li K.K., Oo Y.H., Suresh S., Stamataki Z., Qureshi O., Lalor P.F., Shaw J., Syn W.K. (2013). Monocyte subsets in human liver disease show distinct phenotypic and functional characteristics. Hepatology.

[84] Sica A., Mantovani A. (2012). Macrophage plasticity and polarization: In vivo veritas. J. Clin. Investig..

[85] Gordon S., Martinez F.O. (2010). Alternative activation of macrophages: Mechanism and functions. Immunity.

[86] Sica A., Invernizzi P., Mantovani A. (2014). Macrophage plasticity and polarization in liver homeostasis and pathology. Hepatology.

[87] Wan J., Benkdane M., Teixeira-Clerc F., Bonnafous S., Louvet A., Lafdil F., Pecker F., Tran A., Gual P., Mallat A. (2014). M2 Kupffer cells promote M1 Kupffer cell apoptosis: A protective mechanism against alcoholic and nonalcoholic fatty liver disease. Hepatology.

[88] Xue J., Sharma V., Hsieh M.H., Chawla A., Murali R., Pandol S.J., Habtezion A. (2015). Alternatively activated macrophages promote pancreatic fibrosis in chronic pancreatitis. Nat. Commun..

[89] Proudfoot A.E. (2002). Chemokine receptors: Multifaceted therapeutic targets. Nat. Rev. Immunol..

[90] Kanda H., Tateya S., Tamori Y., Kotani K., Hiasa K., Kitazawa R., Kitazawa S., Miyachi H., Maeda S., Egashira K. (2006). MCP-1 contributes to macrophage infiltration into adipose tissue, insulin resistance, and hepatic steatosis in obesity. J. Clin. Investig..

[91] Conductier G., Blondeau N., Guyon A., Nahon J.L., Rovere C. (2010). The role of monocyte chemoattractant protein MCP1/CCL2 in neuroinflammatory diseases. J. Neuroimmunol..

[92] De Waard V., Bot I., de Jager S.C., Talib S., Egashira K., de Vries M.R., Quax P.H., Biessen E.A., van Berkel T.J. (2010). Systemic MCP1/CCR2 blockade and leukocyte specific MCP1/CCR2 inhibition affect aortic aneurysm formation differently. Atherosclerosis.

[93] Kitade H., Sawamoto K., Nagashimada M., Inoue H., Yamamoto Y., Sai Y., Takamura T., Yamamoto H., Miyamoto K.-I., Ginsberg H.N. (2012). CCR5 plays a critical role in obesity-induced adipose tissue inflammation and insulin resistance by regulating both macrophage recruitment and M1/M2 status. Diabetes.

[94] Wynn T.A., Barron L. (2010). Macrophages: Master regulators of inflammation and fibrosis. Semin. Liver Dis..

[95] Schuppan D., Kim Y.O. (2013). Evolving therapies for liver fibrosis. J. Clin. Investig..

[96] Iredale J., Benyon R., Pickering J., McCullen M., Northrop M., Pawley S., Hovell C., Arthur M. (1998). Mechanisms of spontaneous resolution of rat liver fibrosis. Hepatic stellate cell apoptosis and reduced hepatic expression of metalloproteinase inhibitors. J. Clin. Investig..

[97] Takehara T., Tatsumi T., Suzuki T., Rucker E.B., Hennighausen L., Jinushi M., Miyagi T., Kanazawa Y., Hayashi N. (2004). Hepatocyte-specific disruption of Bcl-xL leads to continuous hepatocyte apoptosis and liver fibrotic responses. Gastroenterology.

[98] Otogawa K., Kinoshita K., Fujii H., Sakabe M., Shiga R., Nakatani K., Ikeda K., Nakajima Y., Ikura Y., Ueda M. (2007). Erythrophagocytosis by liver macrophages (Kupffer cells) promotes oxidative stress, inflammation, and fibrosis in a rabbit model of steatohepatitis: Implications for the pathogenesis of human nonalcoholic steatohepatitis. Am. J. Pathol..

[99] Tacke F., Zimmermann H.W. (2014). Macrophage heterogeneity in liver injury and fibrosis. J. Hepatol..

[100] Seki E., de Minicis S., Inokuchi S., Taura K., Miyai K., van Rooijen N., Schwabe R.F., Brenner D.A. (2009). CCR2 promotes hepatic fibrosis in mice. Hepatology.

[101] Karlmark K.R., Weiskirchen R., Zimmermann H.W., Gassler N., Ginhoux F., Weber C., Merad M., Luedde T., Trautwein C., Tacke F. (2009). Hepatic recruitment of the inflammatory GR1^+^ monocyte subset upon liver injury promotes hepatic fibrosis. Hepatology.

[102] Musso G., Gambino R., Cassader M., Pagano G. (2010). A meta-analysis of randomized trials for the treatment of nonalcoholic fatty liver disease. Hepatology.

[103] Arab J.P., Candia R., Zapata R., Munoz C., Arancibia J.P., Poniachik J., Soza A., Fuster F., Brahm J., Sanhueza E. (2014). Management of nonalcoholic fatty liver disease: An evidence-based clinical practice review. World J. Gastroenterol.

[104] Younossi Z.M. (2008). Review article: Current management of non-alcoholic fatty liver disease and non-alcoholic steatohepatitis. Aliment. Pharmacol. Ther..

[105] Sanyal A.J., Chalasani N., Kowdley K.V., McCullough A., Diehl A.M., Bass N.M., Neuschwander-Tetri B.A., Lavine J.E., Tonascia J., Unalp A. (2010). Pioglitazone, vitamin E, or placebo for nonalcoholic steatohepatitis. N. Engl. J. Med..

[106] Lavine J.E., Schwimmer J.B., van Natta M.L., Molleston J.P., Murray K.F., Rosenthal P., Abrams S.H., Scheimann A.O., Sanyal A.J., Chalasani N. (2011). Effect of vitamin E or metformin for treatment of nonalcoholic fatty liver disease in children and adolescents: The tonic randomized controlled trial. JAMA.

[107] Wang F., Wen T., Chen X.Y., Wu H. (2008). Protective effects of pirfenidone on D-galactosamine and lipopolysaccharide-induced acute hepatotoxicity in rats. Inflamm. Res..

[108] Tsuchiya H., Kaibori M., Yanagida H., Yokoigawa N., Kwon A.H., Okumura T., Kamiyama Y. (2004). Pirfenidone prevents endotoxin-induced liver injury after partial hepatectomy in rats. J. Hepatol..

[109] McCarthy E.M., Rinella M.E. (2012). The role of diet and nutrient composition in nonalcoholic fatty liver disease. J. Acad. Nutr. Diet..

[110] Dongiovanni P., Lanti C., Riso P., Valenti L. (2016). Nutritional therapy for nonalcoholic fatty liver disease. J. Nutr. Biochem..

[111] Eliades M., Spyrou E., Agrawal N., Lazo M., Brancati F.L., Potter J.J., Koteish A.A., Clark J.M., Guallar E., Hernaez R. (2013). Meta-analysis: Vitamin D and non-alcoholic fatty liver disease. Aliment. Pharmacol. Ther..

[112] Rosen C.J. (2011). Vitamin D insufficiency. N. Engl. J. Med..

[113] Rhee E.-J., Kim M.K., Park S.E., Park C.-Y., Baek K.H., Lee W.-Y., Kang M.I., Park S.-W., Kim S.-W., Oh K.W. (2013). High serum vitamin D levels reduce the risk for nonalcoholic fatty liver disease in healthy men independent of metabolic syndrome. Endocr. J..

[114] Bril F., Maximos M., Portillo-Sanchez P., Biernacki D., Lomonaco R., Subbarayan S., Correa M., Lo M., Suman A., Cusi K. (2015). Relationship of vitamin D with insulin resistance and disease severity in non-alcoholic steatohepatitis. J. Hepatol..

[115] Nobili V., Giorgio V., Liccardo D., Bedogni G., Morino G., Alisi A., Cianfarani S. (2014). Vitamin D levels and liver histological alterations in children with nonalcoholic fatty liver disease. Eur. J. Endocrinol..

[116] Barchetta I., Angelico F., del Ben M., Baroni M.G., Pozzilli P., Morini S., Cavallo M.G. (2011). Strong association between non alcoholic fatty liver disease (NAFLD) and low 25(OH) vitamin D levels in an adult population with normal serum liver enzymes. BMC Med..

[117] Kwok R.M., Torres D.M., Harrison S.A. (2013). Vitamin D and nonalcoholic fatty liver disease (NAFLD): Is it more than just an association?. Hepatology.

[118] Roth C.L., Elfers C.T., Figlewicz D.P., Melhorn S.J., Morton G.J., Hoofnagle A., Yeh M.M., Nelson J.E., Kowdley K.V. (2012). Vitamin D deficiency in obese rats exacerbates nonalcoholic fatty liver disease and increases hepatic resistin and toll-like receptor activation. Hepatology.

[119] Li J., Cordero P., Nguyen V., Oben J.A. (2016). The role of vitamins in the pathogenesis of non-alcoholic fatty liver disease. Integr. Med. Insights.

[120] Hoofnagle J.H., van Natta M.L., Kleiner D.E., Clark J.M., Kowdley K.V., Loomba R., Neuschwander-Tetri B.A., Sanyal A.J., Tonascia J. (2013). Vitamin E and changes in serum alanine aminotransferase levels in patients with non-alcoholic steatohepatitis. Aliment. Pharmacol. Ther..

[121] Lavine J.E. (2000). Vitamin E treatment of nonalcoholic steatohepatitis in children: A pilot study. J. Pediatr..

[122] Harrison S.A., Torgerson S., Hayashi P., Ward J., Schenker S. (2003). Vitamin E and vitamin C treatment improves fibrosis in patients with nonalcoholic steatohepatitis. Am. J. Gastroenterol..

[123] Klein E.A., Thompson I.M., Tangen C.M., Crowley J.J., Lucia M.S., Goodman P.J., Minasian L.M., Ford L.G., Parnes H.L., Gaziano J.M. (2011). Vitamin E and the risk of prostate cancer: The selenium and vitamin E cancer prevention trial (select). JAMA.

[124] Ambati R.R., Phang S.M., Ravi S., Aswathanarayana R.G. (2014). Astaxanthin: Sources, extraction, stability, biological activities and its commercial applications—a review. Mar. Drugs.

[125] Kurashige M., Okimasu E., Inoue M., Utsumi K. (1989). Inhibition of oxidative injury of biological membranes by astaxanthin. Physiol. Chem. Phys. Med. NMR.

[126] Guerin M., Huntley M.E., Olaizola M. (2003). Haematococcus astaxanthin: Applications for human health and nutrition. Trends Biotechnol..

[127] Yuan J.P., Peng J., Yin K., Wang J.H. (2011). Potential health-promoting effects of astaxanthin: A high-value carotenoid mostly from microalgae. Mol. Nutr. Food Res..

[128] Kang J., Kim S., Kim H. (2001). Effect of astaxanthin on the hepatotoxicity, lipid peroxidation and antioxidative enzymes in the liver of CCL4-treated rats. Methods Find. Exp. Clin. Pharmacol..

[129] Ikeuchi M., Koyama T., Takahashi J., Yazawa K. (2007). Effects of astaxanthin in obese mice fed a high-fat diet. Biosci. Biotechnol. Biochem..

[130] Ishiki M., Nishida Y., Ishibashi H., Wada T., Fujisaka S., Takikawa A., Urakaze M., Sasaoka T., Usui I., Tobe K. (2013). Impact of divergent effects of astaxanthin on insulin signaling in L6 cells. Endocrinology.

[131] Yang Y., Bae M., Kim B., Park Y.K., Koo S.I., Lee J.Y. (2016). Astaxanthin prevents and reverses the activation of mouse primary hepatic stellate cells. J. Nutr. Biochem..

[132] Yang Y., Kim B., Park Y.K., Koo S.I., Lee J.Y. (2015). Astaxanthin prevents TGFβ1-induced pro-fibrogenic gene expression by inhibiting SMAD3 activation in hepatic stellate cells. Biochim. Biophys. Acta.

[133] Ni Y., Nagashimada M., Zhuge F., Zhan L., Nagata N., Tsutsui A., Nakanuma Y., Kaneko S., Ota T. (2015). Astaxanthin prevents and reverses diet-induced insulin resistance and steatohepatitis in mice: A comparison with vitamin E. Sci. Rep..

[134] Khaire A., Rathod R., Kale A., Joshi S. (2015). Vitamin B12 and ω-3 fatty acids together regulate lipid metabolism in wistar rats. Prostaglandins Leukot. Essent. Fatty Acids.

[135] Deshmukh U., Katre P., Yajnik C.S. (2013). Influence of maternal vitamin B12 and folate on growth and insulin resistance in the offspring. Maternal and Child Nutrition: The First 1000 Days.

[136] Haegele A.D., Gillette C., O’Neill C., Wolfe P., Heimendinger J., Sedlacek S., Thompson H.J. (2000). Plasma xanthophyll carotenoids correlate inversely with indices of oxidative DNA damage and lipid peroxidation. Cancer Epidemiol. Biomark. Prev..

[137] Ni Y., Nagashimada M., Zhan L., Nagata N., Kobori M., Sugiura M., Ogawa K., Kaneko S., Ota T. (2015). Prevention and reversal of lipotoxicity-induced hepatic insulin resistance and steatohepatitis in mice by an antioxidant carotenoid, β-cryptoxanthin. Endocrinology.

[138] Kobori M., Ni Y., Takahashi Y., Watanabe N., Sugiura M., Ogawa K., Nagashimada M., Kaneko S., Naito S., Ota T. (2014). β-Cryptoxanthin alleviates diet-induced nonalcoholic steatohepatitis by suppressing inflammatory gene expression in mice. PLoS ONE.

[139] Rinella M.E., Sanyal A.J. (2015). NAFLD in 2014: Genetics, diagnostics and therapeutic advances in NAFLD. Nat. Rev. Gastroenterol. Hepatol..

[140] Ni X., Wang H. (2016). Silymarin attenuated hepatic steatosis through regulation of lipid metabolism and oxidative stress in a mouse model of nonalcoholic fatty liver disease (NAFLD). Am. J. Transl. Res..

[141] Abenavoli L., Milic N., Peta V., Alfieri F., De Lorenzo A., Bellentani S. (2014). Alimentary regimen in non-alcoholic fatty liver disease: Mediterranean diet. World J. Gastroenterol..

[142] Abenavoli L. (2015). Non-alcoholic fatty liver disease and beneficial effects of dietary supplements. World J. Hepatol..

